# Numerical study on the influence of wall temperature gradient on aerodynamic characteristics of low aspect ratio flying wing configuration

**DOI:** 10.1038/s41598-021-94261-x

**Published:** 2021-08-11

**Authors:** Peng Lin, Xueqiang Liu, Neng Xiong, Xiaobing Wang, Ma Shang, Guangyuan Liu, Yang Tao

**Affiliations:** 1 College of Aerospace Engineering, Nanjing University of Aeronautics and Astronautics, No. 29 Yudao Street, Nanjing, 210016 China; 2 Hunan Lingxiang Maglev Technology Co., Ltd, Changsha, 410004 China; 3 China Aerodynamics Research and Development Center, High Speed Aerodynamic Institute, Mianyang, 621000 China

**Keywords:** Aerospace engineering, Physics

## Abstract

With the aim for a low-aspect-ratio flying wing configuration, this study explores the influence of wall temperature gradient on the laminar and turbulent boundary layers of aircraft surface and determines the effect on the transition Reynolds number and wall friction drag. A four-equation turbulence model with transition mode is used to numerically simulate the flow around the model. The variation of wall friction coefficient, transition Reynolds number, and turbulent boundary layer flow with wall temperature are emphatically investigated. Results show that when the wall temperature increases from 288 to 500 K, the boundary layer transition Reynolds number for the wing section increased by approximately 28% and the surface friction drags decreases by approximately 10.7%. The hot wall enhances the viscous effects of the laminar temperature boundary layer, reduces the Reynolds shear stress and turbulent kinetic energy, and increases the flow stability. However, the velocity gradient and shear stress in the bottom of the turbulent boundary layer decreases, which leads to reduced friction shear stress on the wall surface. Therefore, for the low-aspect-ratio flying wing model, the hot wall can delay the boundary layer transition and reduce the friction drag coefficient in the turbulent region.

## Introduction

The flying wing configuration eliminates the vertical and horizontal tails comparing to conventional configurations, which improves the aerodynamic efficiency and has excellent stealth capability and structural performance. However, the lack of stabilizers and control efficiency has long limited the development of flying wing configuration. With the development of modern control technology and the emergence of new design concepts, the defects of flying wing configuration can be effectively restrained within a certain range, which gradually increases the practicality of the flying wing configuration. The American B-2 long-range bomber is a successful application of high-aspect-ratio flying wing configuration.

**Figure 1:**
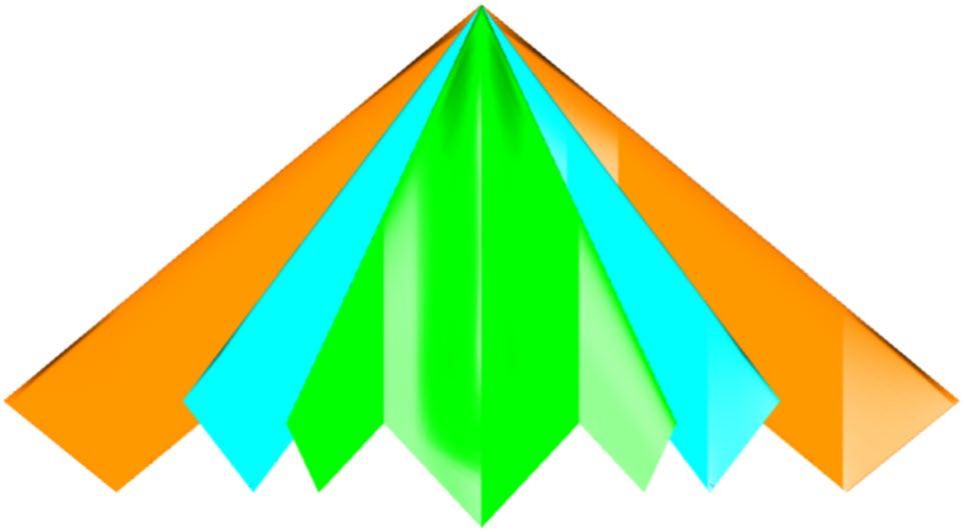
Flying wing configurations with different aspect ratios.

In recent years, countries all over the world are competing to develop unmanned combat air vehicle (UCAV), such as the X-45A/B/C and X-47A/B of the United States (US), “Neurons” developed by many European countries, and Raytheon unmanned aerial vehicle (UAV) of the United Kingdom. Without prior consultation, all these UCAVs have adopted the flying wing configuration with medium-aspect-ratio, which shows its importance. At the same time, Europe and the United States have launched general research models with flying wing configuration characteristics, such as the new control surface model of innovative control effector (ICE) flying wing configuration designed by Lockheed Martin^1,2^, UCAV flying wing configuration series designed by Boeing^3^, and the stability and control configuration(SACCON based on Boeing 1303 and a tailless 53 degree swept angle lamda wing UAV designed ) general flying wing research configuration led by Europe with participation from the United States^4^. Based on the research of these flying wing configurations, the typical flow characteristics of aircraft with similar configurations can be obtained to provide technical support for the development of UCAV. The shock wave drag in transonic and supersonic region can be reduced by decreasing the aspect ratio and increasing the leading edge sweep angle^2,3^. The higher flight speed requirements of future aircraft can promote the development of flying wing configurations toward a smaller aspect ratio, which is also an important direction of future UAV development. At present, the cruise speed of such low-aspect-ratio aircraft is in the high subsonic speed range, and the research on drag reduction is of great significance to the design of the entire aircraft. Figure 1 provides flying wing configurations with different aspect ratios. At present, the layout of flying wing is mainly used in the subsonic range. Because of its high lift drag ratio, the high aspect ratio flying wing is used in transport aircraft for a long time, but its drag divergence Mach number is small, so it is not suitable for high subsonic flight. Because of its large leading edge sweep angle the low aspect flying wing can fly in high subsonic speed and has high endurance efficiency so it is an alternative layout for future fighters.

The friction drag on the aircraft surface can be significantly reduced by delaying the boundary layer transition on the aircraft surface and maintaining the laminar flow as large as possible ^4^. Therefore, how to reduce the surface drag of aircraft through natural laminar flow (NLF) to improve the range and flight efficiency is crucial for aircraft design, and is also an important means to realize the long-term plan of emission reduction in 2050^1, 2^. In the design, the factors affecting the aircraft surface flow state mainly include the pressure gradient in the flow direction, surface heat flow, and surface mass transfer^4^. These factors have a significant effect on the stability of the leading edge laminar flow and are the possible sources of disturbance, especially for aircraft flying with low turbulence. When the wing sweep angle is less than 15°–20°, the unsteady flow direction is the leading cause of the transition, and the NLF can be realized by obtaining the necessary forward pressure gradient through the design of aerodynamic configuration. However, the decrease of aspect ratio means that the leading edge of the aircraft has a large sweep angle; three-dimensional (3D) instability mechanisms are dominant, such as secondary flow and cross-flow instability. In this case, NLF cannot be realized unless active flow control methods are combined.

The influence of wall temperature gradient on laminar flow stability and boundary layer transition is one of the hot spots in aerodynamic research. As early as 1947, Lees^5^ found that wall cooling can stabilize the two-dimensional disturbance in the model boundary layer. In 1976, Boehman^6^ found that wall cooling can stabilize two- and three-dimensional disturbances in transonic flow. In 1980, Potter^7^ summarized the experiments on wall cooling at hyper-sonic speed, and found that in most cases, the transition Reynolds number increases as the wall temperature decreases. Research on the influence of wall temperature on the large sweep angle and three-dimensional flow started relatively late. In 1986, Lin^1^ carried out a numerical study on wall heating of a slender symmetrical blunt body similar to fuselage, and found that heating can reduce friction and total drags. On this basis, Kramer^2^ carried out an experimental study of heating control on different positions of slender blunt body in 1999, and found that the more forward the heating position, the larger the heating area and the greater the drag reduction. Lin and Kramer believed that the change of drag was due to the influence of wall temperature on turbulent boundary layer, but did not point out the mechanism. Subsequently, numerous research on this area has emerged but mainly focused on the hyper-sonic field, or the development of certain disturbances in the boundary layer with a small sweep angle^8–10^. Therefore, the effect of temperature gradient of flying wing configuration with small aspect ratio on boundary layer transition needs further exploration^11^.

The present study mainly focuses on the effect of model surface temperature gradient on the flying wing with large sweep angle and small aspect ratio. A four-equation turbulence model with transition mode is used to simulate the flow of flying wing calibration model with small aspect ratio. The variations of wall friction coefficient, transition Reynolds number, and turbulent boundary layer flow with wall temperature are emphatically investigated.

## Numerical method

We use a CFD solver called PMB3D for following simulation. PMB3D is developed by CARDC, based on structured grid and parallel MPI. It is suitable for numerical simulation of low-speed, subsonic, transonic, supersonic and hypersonic flows, and is competent for transonic transition problems. PMB3D has been verified by a large number of standard models and cases, with good convergence and high accuracy.

### Governing equations and $\gamma -{Re}_{\theta }$ transition model

The three-dimensional Reynolds-averaged Navier–Stokes equations are used as the governing equations to describe the physical phenomena, which can be expressed by the finite volume form:

1 $$\frac{\partial }{\partial \mathbf{t}}{\int }_{{\varvec{\Omega}}}\mathbf{W}\mathbf{d}{\varvec{\Omega}}+{\oint }_{\partial{\varvec{\Omega}}}\left[\mathbf{F}-{\mathbf{F}}_{\mathbf{v}}\right]\mathbf{d}\mathbf{S}=0$$

where **W** is the vector of conservative variables; **F** and **F**_**v**_ are in-viscid and viscous flux vectors, respectively; ${\varvec{\Omega}}$ is the control volume with the boundary $\partial{\varvec{\Omega}}$; and **dS** is the infinitesimal face vector.

Accurate prediction of transition position is one of the key technologies for aircraft performance prediction. At present, the two most commonly used methods in engineering design are the **e**^**N**^ and ${\varvec{\gamma}}-{{\varvec{R}}{\varvec{e}}}_{{\varvec{\theta}}}$ transition model^11–14^. A semi-empirical method, **e**^**N**^ is mainly based on linear stability theory and experimental data^15,16^. The value of **N** is greatly affected by the environment. The relationship between turbulence degree and **N** is mainly determined by experiments, which is difficult to popularize to the three-dimensional flow^17,18^.

In this study, the ${\varvec{\gamma}}-{{\varvec{R}}{\varvec{e}}}_{{\varvec{\theta}}}$ transition model developed by Menter et al. is used for transition calculation ^18^. This model does not seek to simulate the specific and complex physical process of transition, but rather to control the generation of intermittent factors in the boundary layer through empirical correlation function and transition momentum Reynolds number. Thus, the location of transition is determined^19^.

The transport equation of intermittent factor $\gamma$ is defined as:

$$\frac{\partial \left(\rho \gamma \right)}{\partial t}+\frac{\partial \left(\rho {U}_{j}\gamma \right)}{\partial {x}_{j}}={P}_{\gamma 1}-{E}_{\gamma 1}+{P}_{\gamma 2}-{E}_{\gamma 2}+\frac{\partial }{\partial {x}_{j}}[(\upmu +\frac{{\mu }_{t}}{{\sigma }_{\gamma }})\frac{\partial \gamma }{\partial {x}_{j}}]$$

The transition source term is defined as:

$${P}_{\gamma 1}={C}_{\alpha 1}{F}_{length}\rho S{[\gamma {F}_{onset}]}^{{C}_{\gamma 3}}$$

$${E}_{\gamma 1}={C}_{e1}{P}_{\gamma 1}\gamma$$

S is the modulus of mean strain rate tensor, ${F}_{length}$ is the length of transition zone, ${C}_{\alpha 1}$ and ${C}_{e1}$ are model coefficients, the destruction/relaminarization term is defined as:

$${P}_{\gamma 2}={C}_{\alpha 2}\mathrm{\rho \Omega \gamma }{F}_{turb}$$

$${E}_{\gamma 2}={C}_{e2}{P}_{\gamma 2}\gamma$$

where $\Omega$ is the modulus of the mean vorticity tensor.

The transition is controlled by the following equations:

$${Re}_{V}=\frac{\uprho {y}^{2}S}{\mu }$$

$${R}_{T}=\frac{\mathrm{\rho k}}{\mu \omega }$$

$${F}_{onset1}=\frac{{Re}_{v}}{{2193Re}_{\theta c}}$$

$${F}_{onset2}=\mathrm{min}(\mathrm{max}\left({F}_{onset1},{F}_{onset1}^{4}\right),2.0)$$

$${F}_{onset3}=\mathrm{max}(1-{(\frac{{R}_{T}}{25})}^{3},0)$$

$${F}_{onset}=\mathrm{max}({F}_{onset2}-{F}_{onset3},0)$$

$${F}_{turb}={e}^{({\frac{{R}_{T}}{4})}^{4}}$$

$y$ is the minimum distance from the wall, ${Re}_{\theta c}$ is the critical momentum thickness Reynolds number.

The model coefficients are defined as follows:

$${C}_{\alpha 1}=2;{C}_{e1}=1;{C}_{\alpha 2}=0.06;{C}_{e2=50};{C}_{\gamma 3}=0.5;{\sigma }_{\gamma }=1.0$$

The transport equation for the transition momentum thickness Reynolds number  ${\tilde{Re }}_{\theta t}$ is defined as:

$$\frac{\partial (\rho {\tilde{Re }}_{\theta t})}{\partial t}+\frac{\partial ({\rho {U}_{j}\tilde{Re }}_{\theta t})}{\partial {x}_{j}}={P}_{\theta t}+\frac{\partial }{\partial {x}_{j}}[{\sigma }_{\theta t}(\mu +{\mu }_{t})\frac{{\tilde{Re }}_{\theta t}}{\partial {x}_{j}}]$$

The production term is defined as:

$${P}_{\theta t}={C}_{\theta t}\frac{\rho }{t}({Re}_{\theta t}-{\tilde{Re }}_{\theta t})(1.0-{F}_{\theta t})$$

$$\mathrm{t}=\frac{500\mu }{\rho {U}^{2}}$$

$${F}_{\theta t}=\mathrm{min}(\mathrm{max}\left(\begin{array}{c}{F}_{wake}{e}^{{\left(-\frac{y}{\delta }\right)}^{4}},1.0-\\ {(\frac{\gamma -1/50}{1.0-1/50})}^{2}\end{array}\right),1.0)$$

$${\theta }_{BL}=\frac{{\tilde{Re }}_{\theta t}\mu }{\rho U}$$

$${\delta }_{BL}=\frac{15}{2}{\theta }_{BL}$$

$${\delta =\frac{50\Omega y}{U}\delta }_{BL}$$

$${Re}_{\omega }=\frac{\rho \omega {y}^{2}}{\mu }$$

$${F}_{wake}={e}^{-{(\frac{{Re}_{\omega }}{1E+5})}^{2}}$$

Other coefficients are defined as:

$${C}_{\theta t}=0.03;{\sigma }_{\theta t}=2.0$$

### Verification of transition model

**Figure 2:**
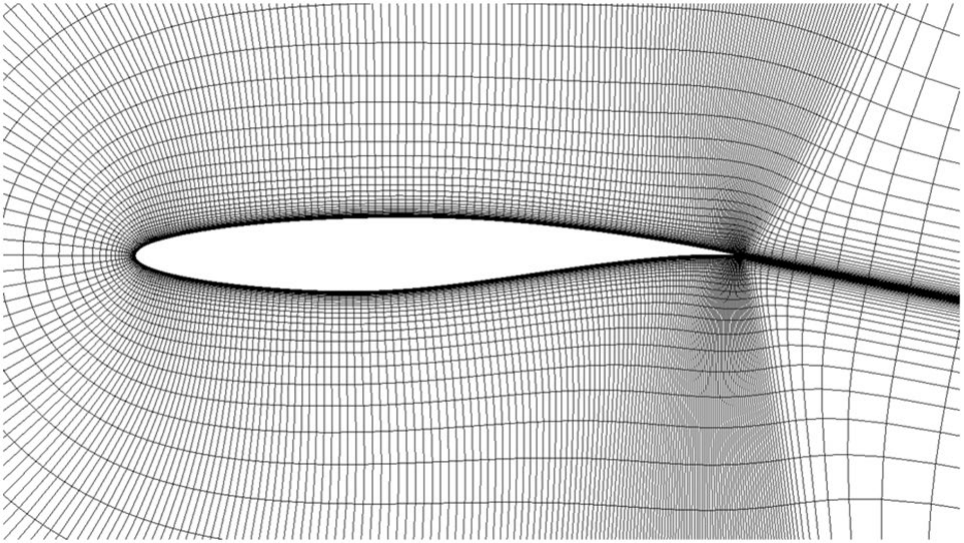
Computational grid of RAE2822.

**Figure 3:**
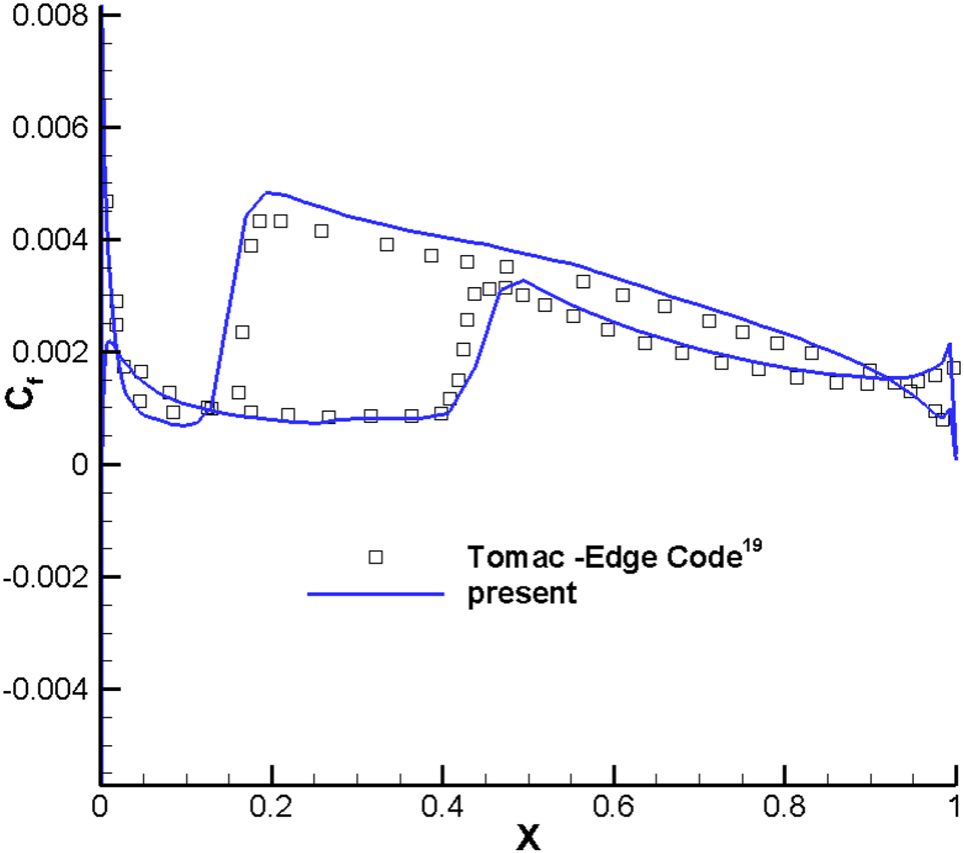
Skin friction coefficient distribution of RAE2822.

**Figure 4:**
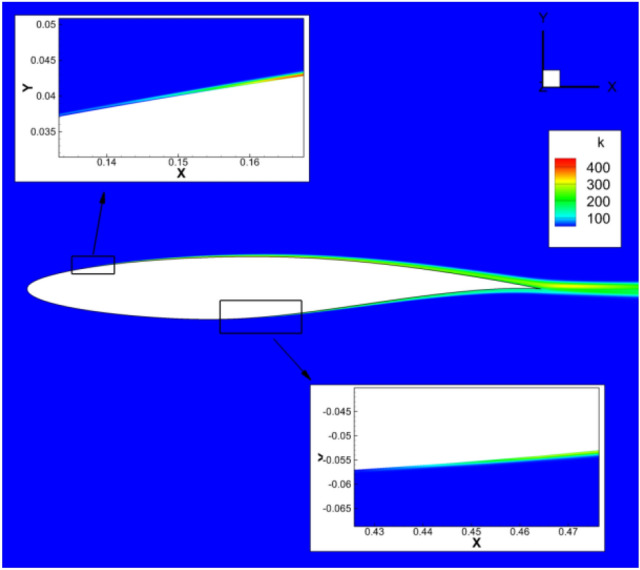
Kinetic energy *k* distribution contour.

**Figure 5:**
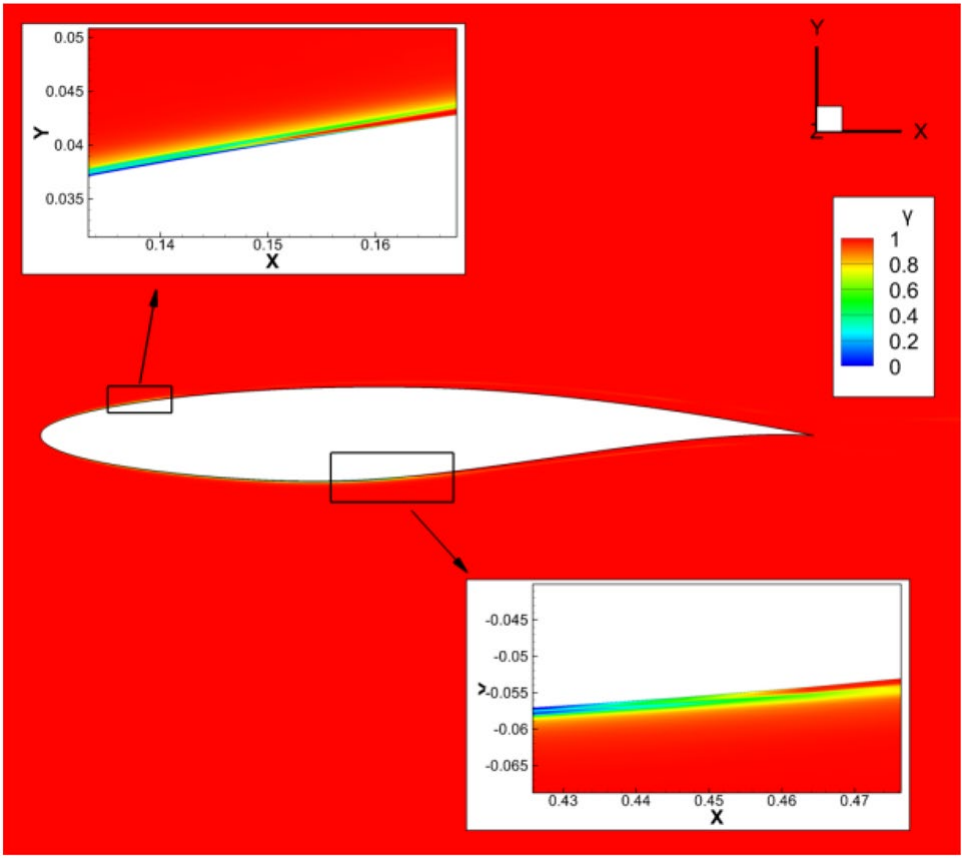
Intermittency factor *γ* distribution contour.

The RAE2822 airfoil is selected to validate the transition prediction of the program in compressible flow calculation. Table 1 shows the calculation conditions and the reference length is the mean aerodynamic chord length of the low aspect ratio flying wing. The turbulence intensity and turbulent viscosity ratio of the incoming flow are consistent with the experimental conditions ^19^. Two dimensional Structural grids are adopted with the grid size 369 × 97. The minimum distance of the first layer grid near the object plane is 5 × 10^–6^, and *y*^+^ < 1 is guaranteed. Figure 2 shows the computational grid and Fig. 3 shows the comparison between the calculated and the experimentally measured transition position. Figure 4 and 5 are the distribution contour of kinetic energy *k* and intermittency factor *γ*.

**Table 1:** Simulated parameters for the test case.

	Reynolds number *Re*	Mach number *M*	Angle of attack *a*	Turbulent viscosity ratio	Turbulence Intensity (%)
RAE2822	1E + 07	0.6	2	10	0.3

### Models and grids

**Figure 6:**
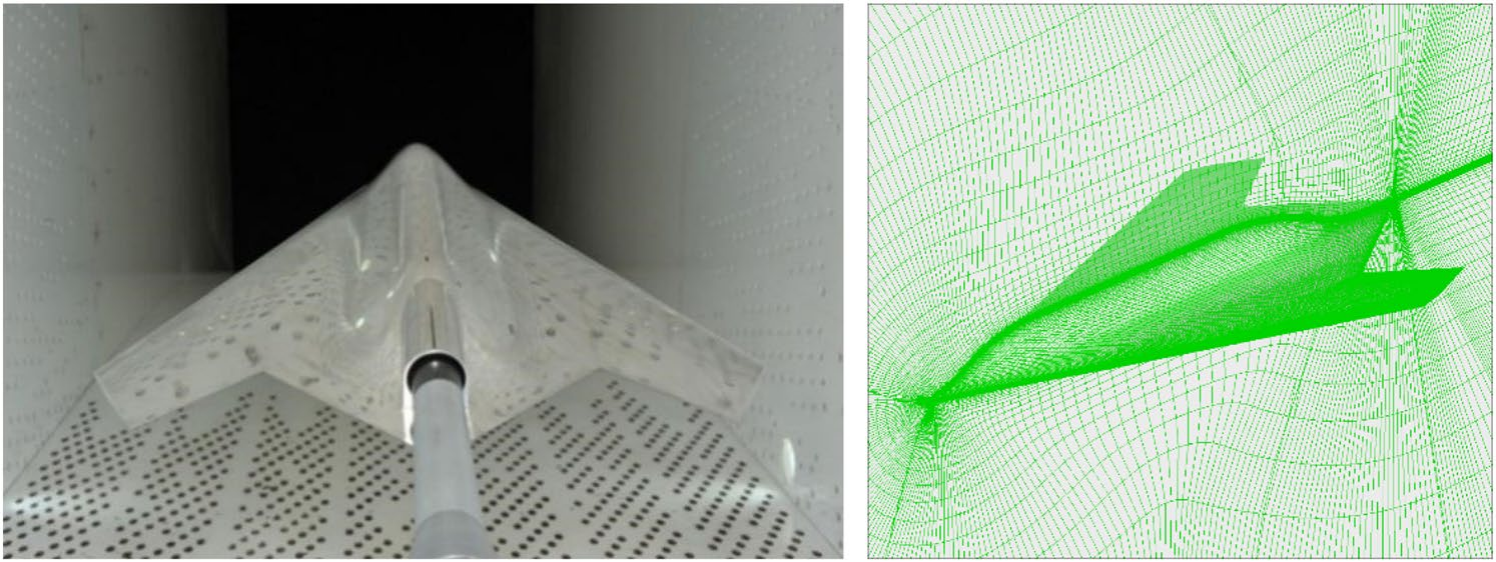
Test model and computational grid.

The low aspect ratio fly wing model adopted in this study is a delta wing configuration with sweep angle of 65° and reference area of 0.234 m^2^. Both the longitudinal and Reynolds number reference lengths are mean aerodynamic chord length, which is 0.5032 m, reference center of gravity is located in 45% b_A_ from the model nose, here b_A_ is the length of the model, calculated Mach number is 0.9 corresponding to cruise state, and the Reynolds number is 8.7 × 10^6^. The numerical calculations of the above parameters are consistent with the wind-tunnel test. Structural grid is used for calculation with approximately 8 million grid points. Figure 6 shows the calculation model and computational grid.

**Figure 7:**
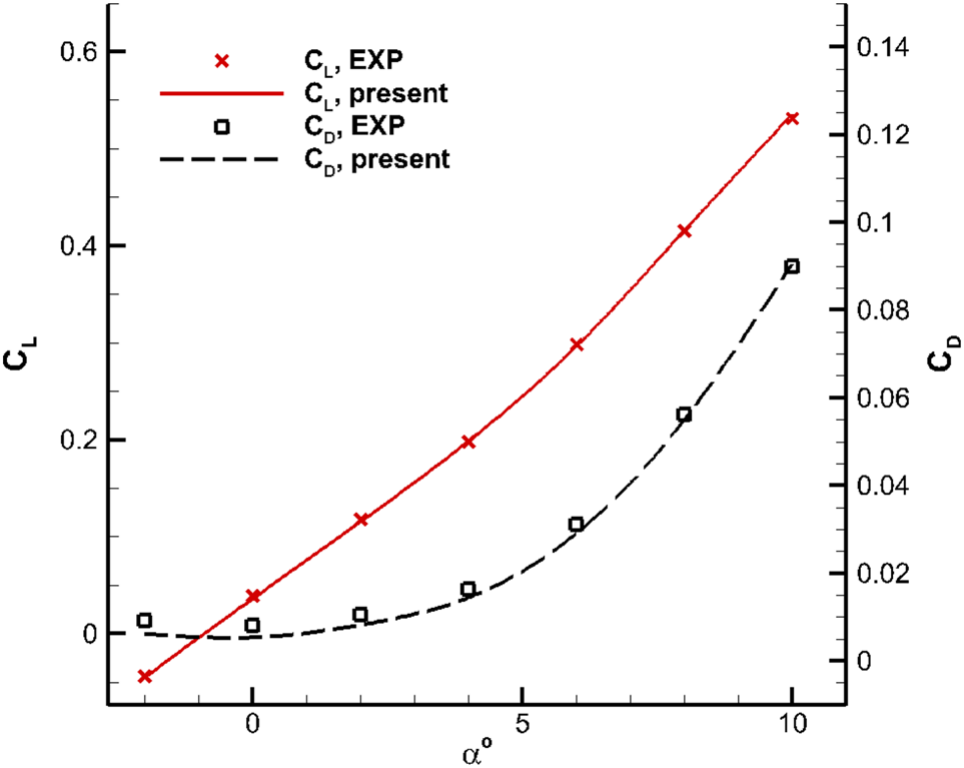
Comparison of CL and CD (M = 0.9).

To further validate the numerical method, the aerodynamic coefficients *C*_*L*_ and *C*_*D*_ for angles of attack ranged between -2° and 10° is calculated and given in Fig. 7.

## Calculation results analysis

### Influence of temperature gradient on aerodynamic characteristics

The influence of wall temperature gradients on the aerodynamic characteristics of the test model is evaluated by defining dimensionless temperature as ${\mathbf{T}}_{\mathbf{w}}^{\mathbf{*}}$ and its expression is ${\mathbf{T}}_{\mathbf{w}}^{\mathbf{*}}={\mathbf{T}}_{\mathbf{w}}/{\mathbf{T}}_{\mathbf{a}\mathbf{w}}$, where ${\mathbf{T}}_{\mathbf{w}}$ is the wall temperature and ${\mathbf{T}}_{\mathbf{a}\mathbf{w}}$ is the adiabatic wall temperature. According to the boundary layer of two-dimensional model, the wall temperature of the laminar and turbulent flow regions can be respectively estimated as follows:

2 $$\frac{{\mathbf{T}}_{\mathbf{a}\mathbf{w}}}{{\mathbf{T}}_{\mathbf{e}}}=\left(1+\mathbf{r}\cdot \frac{\mathbf{r}-1}{2}{\mathbf{M}}_{\mathbf{e}}^{2}\right)$$

${\mathbf{r}}_{\mathbf{l}\mathbf{a}\mathbf{m}}=0.85$ and ${\mathbf{r}}_{\mathbf{t}\mathbf{u}\mathbf{r}\mathbf{b}}=0.90$ in the laminar and turbulent flow regions, respectively. For **Ma** = 0.90, **T**_**e**_ = 254 K, the adiabatic wall temperature is ${\mathbf{T}}_{\mathbf{a}\mathbf{w}}$=291 K.

**Figure 8:**
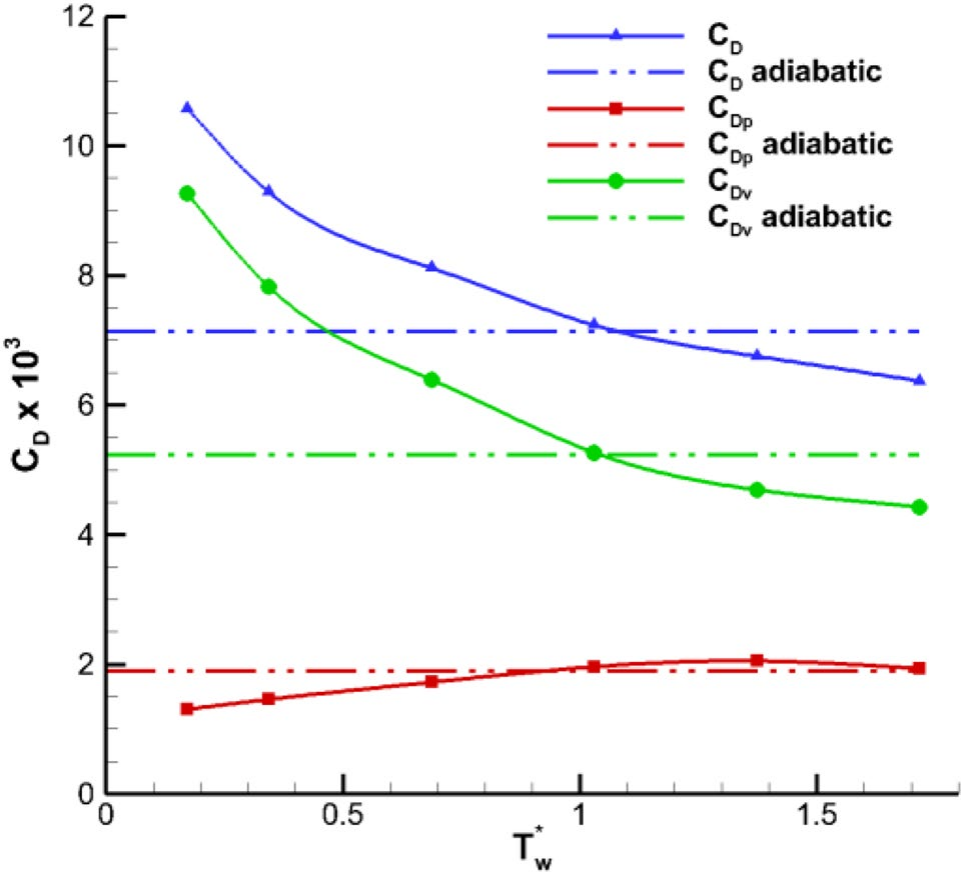
Influence of temperature gradient on model drag coefficients (*M* = 0.9, *α* = 2°).

**Figure 9:**
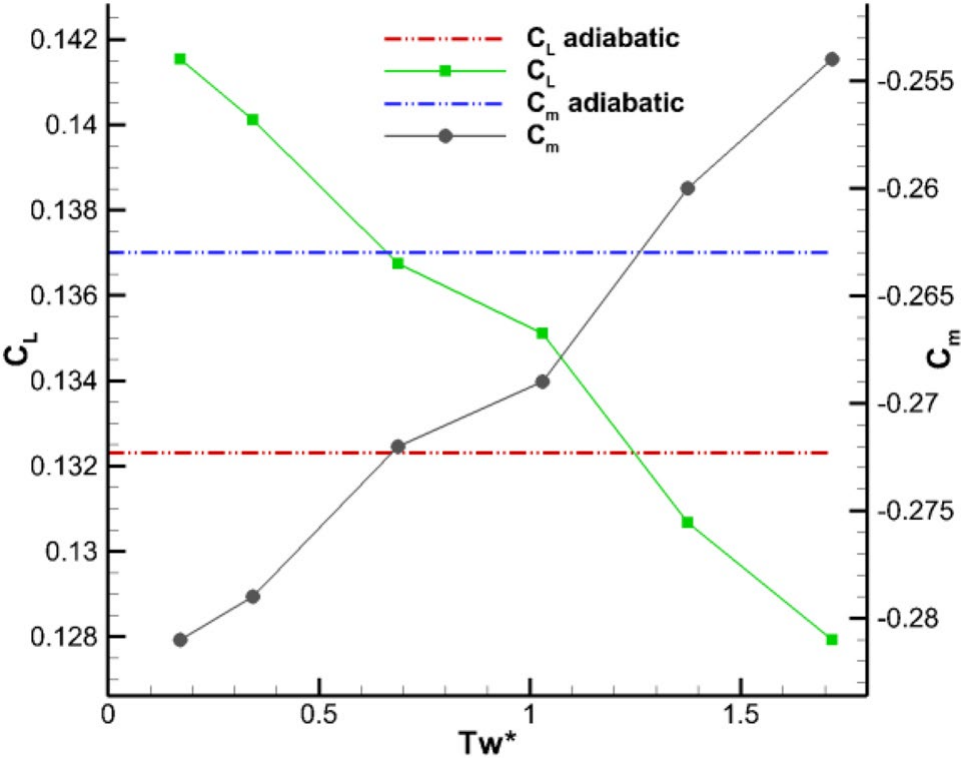
Influence of wall temperature gradient on model lift and pitching moment coefficients (*M* = 0.9, *α* = 2°).

Figure 8 shows the model drag coefficient ${\mathbf{C}}_{\mathbf{D}}$ variation with the model surface temperature gradients, here ${\mathbf{C}}_{\mathbf{D}\mathbf{p}}$ is pressure drag coefficient, ${\mathbf{C}}_{\mathbf{D}\mathbf{v}}$ is viscous drag coefficient. When the wall temperature increases from the adiabatic temperature of 291 K to 500 K, the friction drag decreases by approximately 9.6% and shows good drag reduction effect. From Fig. 8, the wall temperature gradient has an opposite effect on the friction and differential pressure drags. The specific performance is that with the increase of wall temperature, the viscous drag gradually decreases while the differential pressure drag slowly increases with small amplitude. Figure 9 shows the influence of wall temperature gradient on lift and pitching moment coefficient of the entire aircraft. With the increase of wall temperature, the lift and pitching moment varied linearly. According to Eq. (2), the temperature difference between underflow and the turbulent flow regions is about 1.8 K under adiabatic conditions. However, when the wall temperature is close to the static temperature of the incoming flow, its aerodynamic characteristics are basically consistent with the results of adiabatic wall. Therefore, when the wall temperature is close to the static temperature of incoming flow, the wall heat transfer exerts a small aerodynamic influence.

### Influence of temperature gradient on flow characteristics of model surface

**Figure 10:**
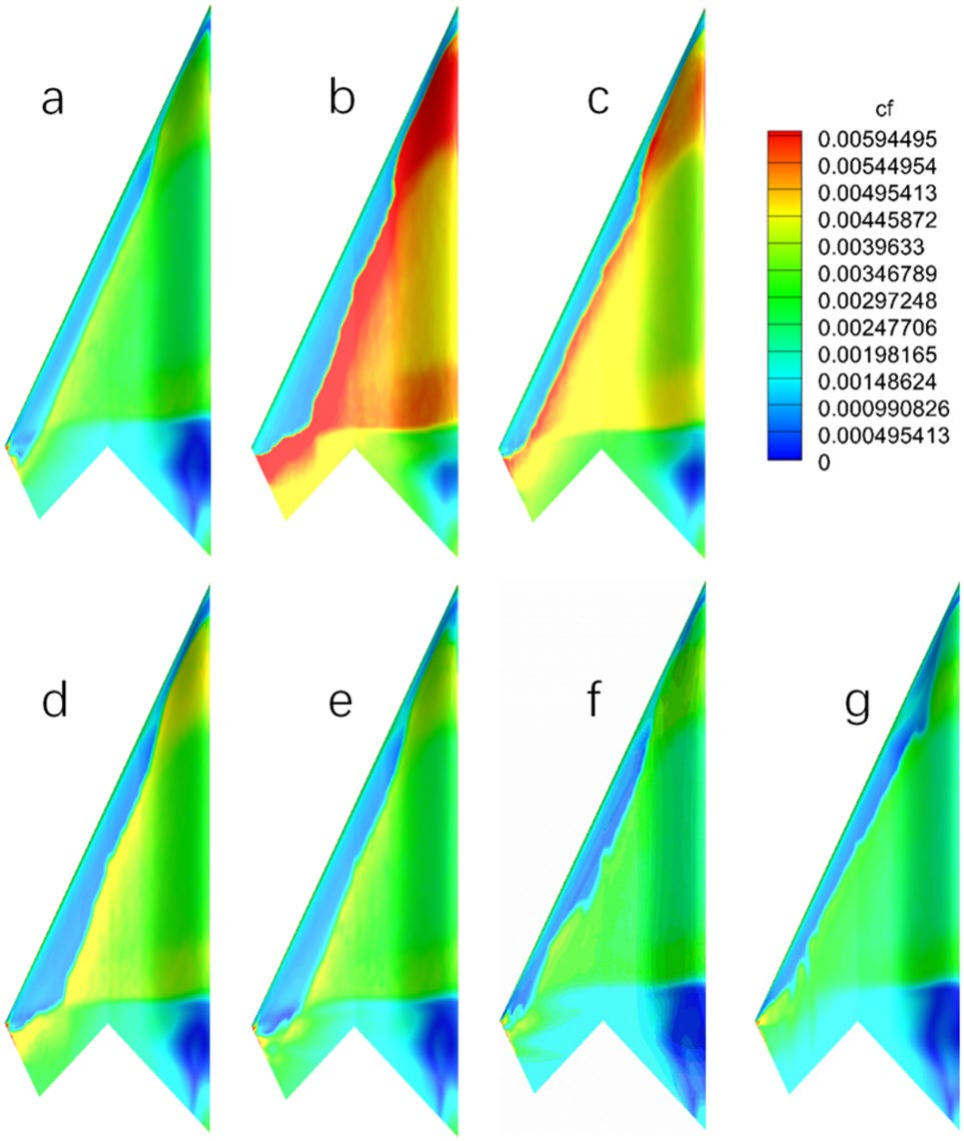
Friction coefficient distributions on the upper surface of flying wing model with wall temperature (*M* = 0.9, α = 2°): **(a)** adiabatic wall; **(b)** ${T}_{w}^{*}=$ 0.172; **(c)** ${T}_{w}^{*}=$ 0.344; **(d)** ${T}_{w}^{*}=$ 0.687; **(e)** ${T}_{w}^{*}=$ 1.031; **(f)** ${T}_{w}^{*}=$ 1.375; **(g)** ${T}_{w}^{*}=$ 1.718.

**Figure 11:**
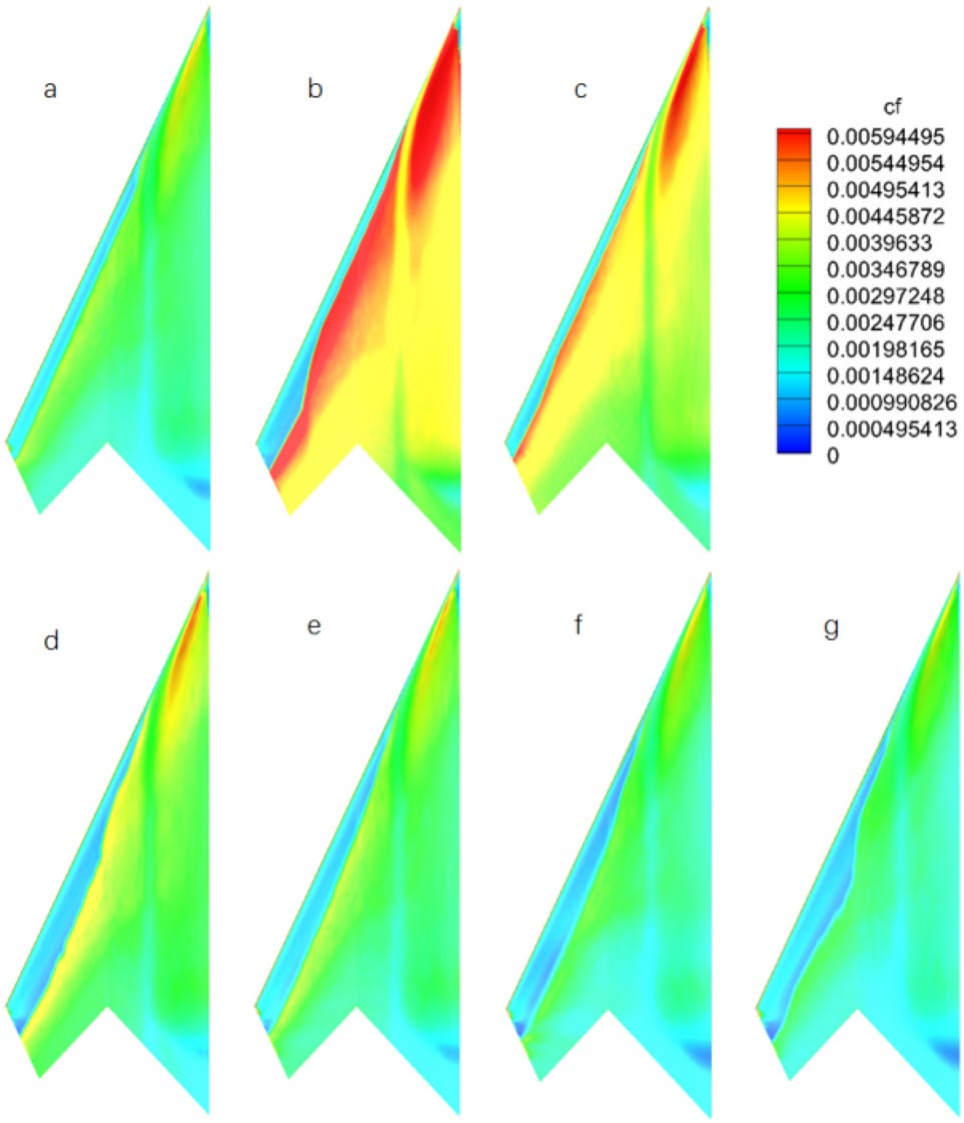
Friction coefficient distributions on the lower surface of flying wing model with wall temperature (*M* = 0.9, α = 2°): **(a)** adiabatic wall; **(b)** ${T}_{w}^{*}=$ 0.172; **(c)** ${T}_{w}^{*}=$ 0.344; **(d)** ${T}_{w}^{*}=$ 0.687; **(e)** ${T}_{w}^{*}=$ 1.031; **(f)** ${T}_{w}^{*}=$ 1.375; **(g)** ${T}_{w}^{*}=$ 1.718.

The influence of temperature gradient on the surface flow characteristics is mainly reflected in the location of the boundary layer transition and friction drag in turbulent boundary layer. Figures 10 and 11 show the contours of the friction stress coefficient distribution on the upper and lower surfaces for different temperature gradients. Under various wall temperature gradients, the laminar flow areas at the leading edge of the model are small because the model has a large sweep angle of 65°. With the simultaneous change of wall temperature gradient, the laminar flow area and the friction coefficient of the model surface change according to a certain law. The flow in the middle wing section is the main concern in the analysis to reduce the influence of the fuselage flow with large thickness on the flying wing. On the adiabatic wall, the laminar flow range of the upper wing decreases at lower wall temperature. The hot wall is opposite to the cold wall, the transition region moves backward and the laminar flow range increases. While the lower airfoil is under the influence of the forward pressure gradient, which is similar to that of the upper airfoil due to the wall temperature gradient, and the effect of laminar flow range is more obvious. Temperature gradient not only affects the transition behavior of the laminar boundary layer, but also affects the friction coefficient in turbulent region, which shows apparent variation law with wall temperature. Turbulent flow after transition in the case of cold wall has a larger friction drag coefficient and smaller friction drag in the case of hot wall.

### Influence of temperature gradient on turbulent boundary layer characteristics

The shear stress and friction coefficient are defined as follows:

3 $${{\varvec{\uptau}}}_{\mathbf{w}}={\varvec{\upmu}}\frac{\mathbf{d}\mathbf{u}}{\mathbf{d}\mathbf{y}}{|}_{\mathbf{y}=0},{\mathbf{c}}_{\mathbf{f}}=\frac{{{\varvec{\uptau}}}_{\mathbf{w}}}{0.5{{\varvec{\uprho}}}_{\mathbf{e}}{\mathbf{u}}_{\mathbf{e}}^{2}}$$

The viscosity of air can be simply evaluated using Sutherland law:

4 $${\varvec{\upmu}}={{\varvec{\upmu}}}_{\mathbf{r}\mathbf{e}\mathbf{f}}\times [{\mathbf{T}}^{1.5}/(\mathbf{T}+110)]\sim {\mathbf{T}}^{0.76}$$

where ${{\varvec{\upmu}}}_{\mathbf{r}\mathbf{e}\mathbf{f}}=1.45\times {10}^{-6}$ is the air viscosity coefficient when **T = 288 K.**

The calculation method of incoming flow density ρ is as follows:

5 $${\varvec{\rho}}={\varvec{p}}/(287\times {\varvec{T}})\sim 1/{\varvec{T}}$$

When the pressure gradient is small, the velocity ${{\varvec{U}}}_{{\varvec{e}}}$ outside the boundary layer can be considered as a constant with small flow direction range in the region where the curvature of the model object plane changes little. In this case, $du/dy$ in the laminar region is inversely proportional to the density change. That is, for ${{\varvec{\tau}}}_{{\varvec{w}}}\sim {{\varvec{T}}}^{0.76}\times {{\varvec{T}}}^{-1}\sim {{\varvec{T}}}^{-0.24}$, the shear stress and Nusselt number of the object plane decrease with the increase of the temperature of the object plane.

**Figure 12:**
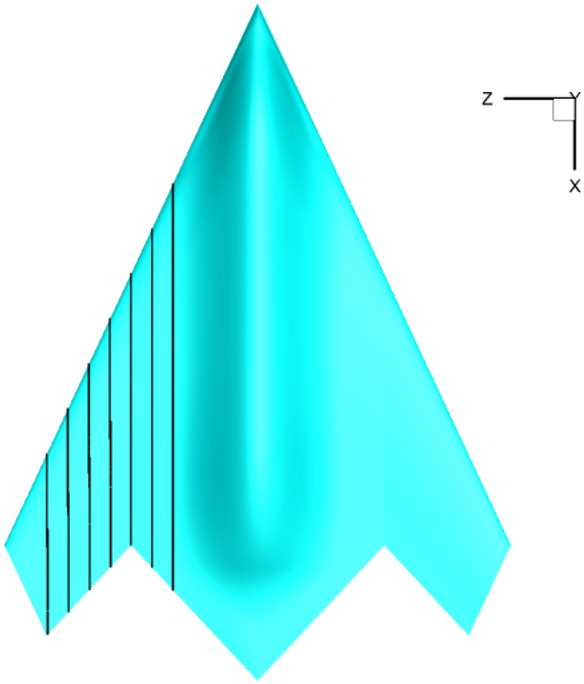
Schematic diagram of span-wise section position.

**Figure 13:**
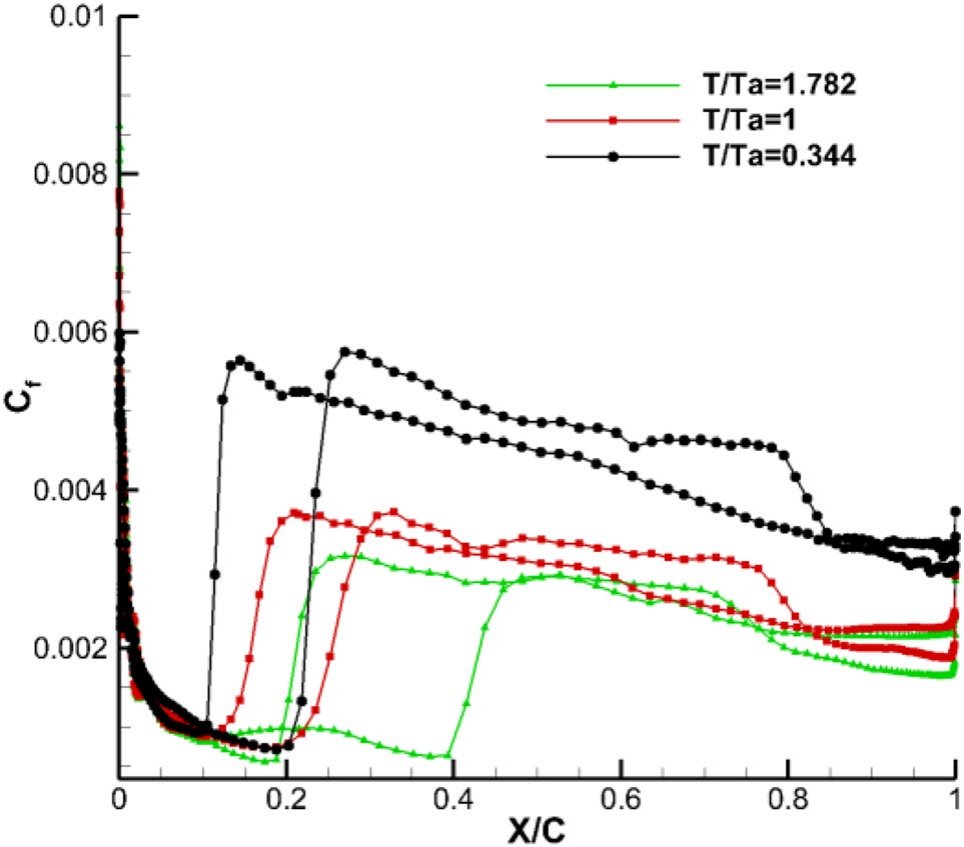
Evolution of friction coefficient, wing section (z = 0.175) at design point (M = 0.9, α = 2.0°).

Figure 12 shows a schematic diagram of the span-wise section positions, on which the skin friction coefficient distributions are computed, the spanwise region is *z* = 0.1–0.25 with interval of $\Delta z=0.025$. Figure 13 shows the distribution of friction stress coefficient at span-wise position of wing *z* = 0.175, from which the influence of temperature gradient is shown more clearly. The calculation results of adiabatic wall are the closest to those under the condition ${\mathbf{T}}_{\mathbf{w}}^{\mathbf{*}}$=1, and only a slight difference is caused by the fact that the wall temperature under adiabatic wall is not completely equal to the static temperature of incoming flow. In the laminar flow region, the friction coefficient is not significantly affected by the wall temperature gradient, but clearly decreases as the wall temperature increases after transition. This finding is consistent with the change in total drag mentioned in “Influence of temperature gradient on aerodynamic characteristics”.

**Figure 14:**
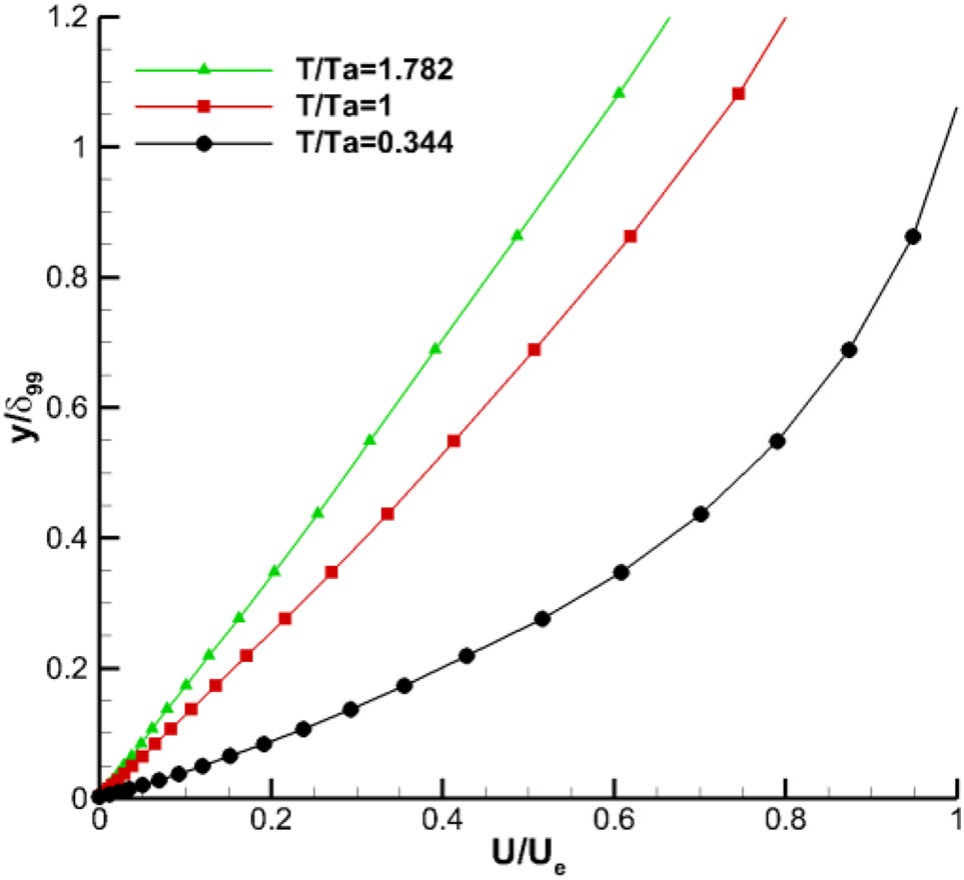
Laminar boundary-layer profiles at z = 0.175 and x/c = 0.085.

The influence of temperature gradient on the boundary layer is analyzed using the boundary layer thickness and the outflow velocity ${\mathbf{U}}_{\mathbf{e}}$ at the entrance wall. Compared with the laminar boundary layer, the turbulent boundary layer has a greater velocity profile. Figure 14 shows the velocity profile at position of *z* = 0.175 and *x/c* = 0.085. Combined with the wall friction coefficient distribution in Fig. 13, the surface flows of the model at this position are laminar and $\frac{du}{dy}{|}_{y=0}$ gradually decreases as the wall temperature increases. From Eq. (4), the viscosity coefficient $\mu$ increases gradually with the increase of temperature. The combined effect indicated that the final wall friction coefficient is basically unchanged.

**Figure 15:**
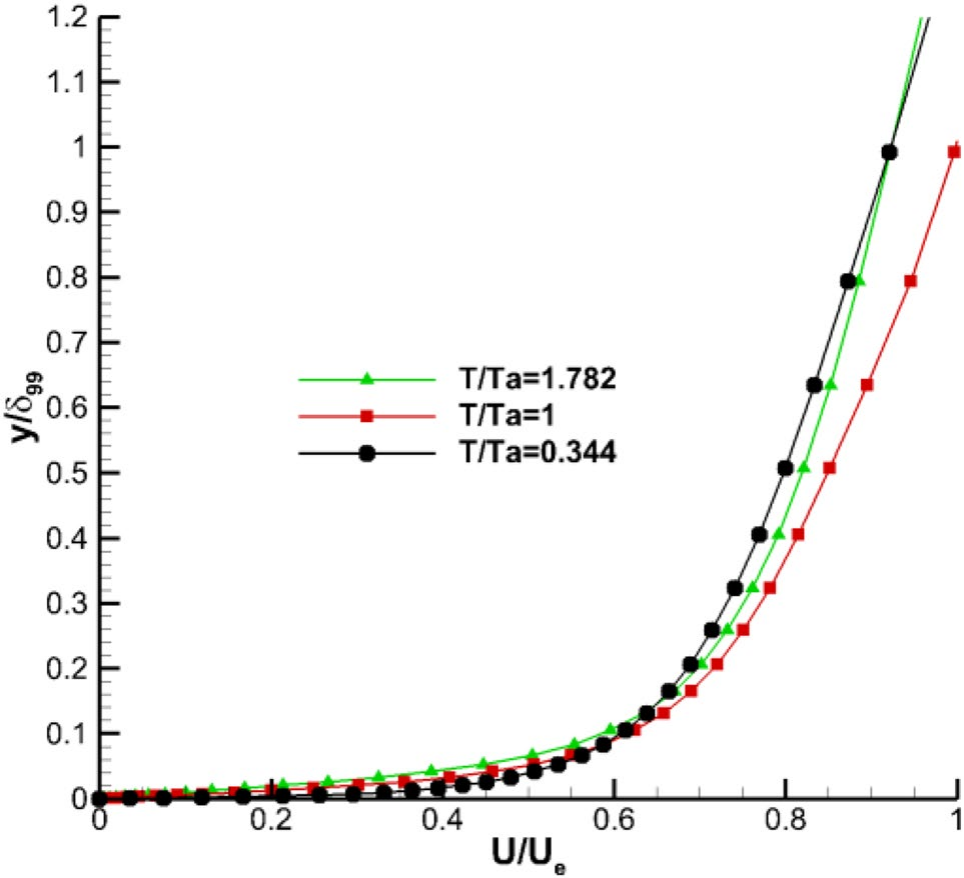
Turbulence boundary-layer profiles at z = 0.175 and x/c = 0.421.

Figure 15 is the velocity pattern at the position **z** = 0.175 and **x/c** = 0.421. Combined with the wall friction coefficient distribution in Fig. 8, the surface flow of the model is turbulent at this position. The diagram shows that the hot wall reduces the velocity gradient and increases the viscosity coefficient at the wall. Given that the velocity gradient near the wall further decreases, the viscous shear stress ${\varvec{\uptau}}$ near the wall also decreases according to Eq. (3). Therefore, the hot wall causes the model wall friction stress ${{\varvec{\uptau}}}_{\mathbf{w}}$ to decrease, and the the wall friction coefficient ${\mathbf{c}}_{\mathbf{f}}$ also decreases. The analysis results from the velocity pattern are consistent with the distribution of friction drag of the flow direction wall in Fig. 10.

**Figure 16:**
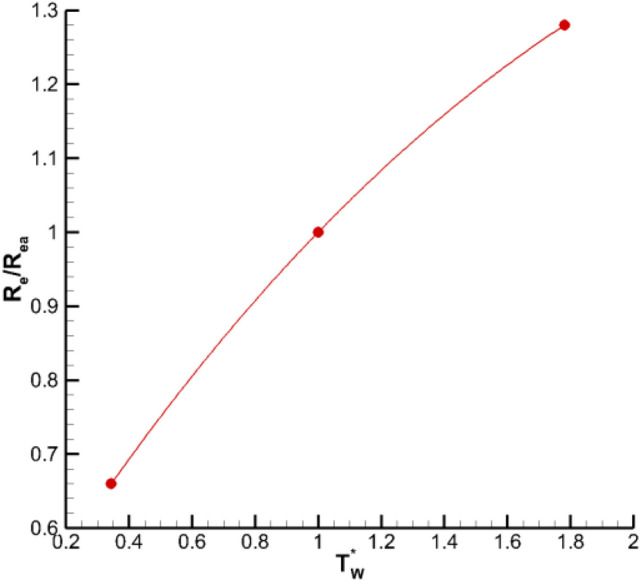
Variation of transition Reynolds numbers for *z* = 0.175 section.

Small disturbances often occur near the wall of the laminar flow. If the disturbance increases with time, the flow becomes unstable and the laminar flow triggers the transition into turbulence. For the downstream-placed mode, with the development of flow, the transition occurs when the boundary layer reaches the critical Reynolds number. Wall temperature gradient leads to the temperature gradient in the boundary layer, which affects air viscosity coefficient and density, and thereby affects the boundary layer transition. Figure 16 shows the change of the model transition Reynolds number at different temperatures. Wall heating increases the transition Reynolds number and delays the transition. When the control temperature increases from 292 to 500 K, the transition Reynolds number increases by approximately 28%.

The influence of temperature on laminar boundary layer was investigated. *η* is defined in Eq. (6). Heating causes a temperature gradient in the boundary layer with high temperature near the wall and low temperature away from the wall. This result further leads to viscous stratification with higher air viscosity coefficient and density stratification with lower density near the wall in the boundary layer.

6 $${\varvec{\upeta}}=\mathbf{y}\sqrt{\frac{{\varvec{\uprho}}\mathbf{V}}{{\varvec{\upmu}}\mathbf{x}}}$$

**Figure 17:**
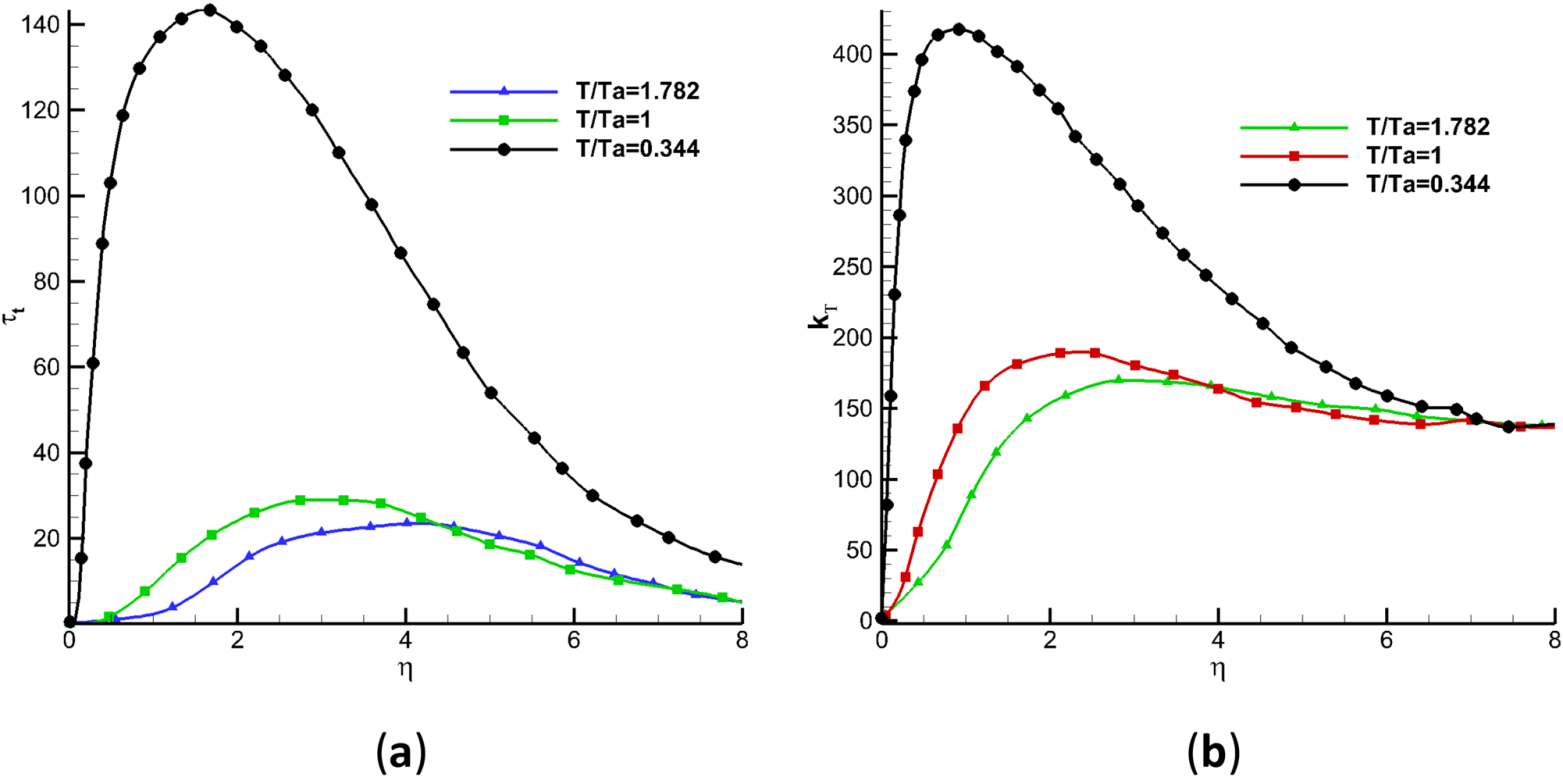
Distribution laws of Reynolds shear stress and turbulent kinetic energy of boundary layer: **(a)** Reynolds shear stress; **(b)** turbulent kinetic energy.

In the turbulent flow, apart from the movement in the mainstream direction, fluid particles also pulse in different directions. Generally, Reynolds shear stress and turbulent kinetic energy can be used to represent the influence of pulsations on time-averaged flow. Figure 17 shows the distribution of Reynolds shear stress ${{\varvec{\uptau}}}_{\mathbf{t}}$ and turbulent kinetic energy ${\mathbf{k}}_{\mathbf{t}}$ in the temperature boundary layer at the same position. Higher wall temperature reduces Reynolds shear stress and turbulent kinetic energy in the boundary layer, that is, the hot wall suppresses the tangential and positive pulsation and stabilizes the flow. Therefore, the wall heating control delays the transition from laminar to turbulent flow, increases the transition Reynolds number and enlarges the laminar flow region. This result is consistent with the distribution laws of macroscopic aerodynamic force and pressure reflected in Figs. 10 and 11.

## Conclusions

In the range of high subsonic velocity, a four-equation transition model with transition mode is used to numerically simulate the effect of wall temperature gradient on the boundary layer on a low-aspect-ratio flying wing configuration model. The effect and mechanism of wall temperature on aerodynamic coefficient, surface flow pattern, and wall friction are analyzed. The main conclusions are as follows:

(1) Higher wall temperature can increase the transition Reynolds number mainly because the higher temperature model surface can increase the viscosity coefficient of the fluid in the laminar boundary layer thus increasing the viscosity effect and reducing the Reynolds shear stress and turbulent kinetic energy as well as restraining the pulsation and increasing the flow stability.

(2) The heated wall reduces the velocity gradient in the bottom of the turbulent region of the model and the viscous shear stress near the wall, thus reducing the friction stress and coefficient at the wall.

(3) Wall heating can delay boundary layer transition, enlarge laminar flow area, and reduce friction drag in the turbulent flow area, thus reducing model drag. The higher the wall temperature, the smaller the model drag.

(4) Wall cooling control can advance the transition, reduce the transition Reynolds number of the wing section and increase the friction drag. The lower the wall temperature means lower transition Reynolds number and the higher drag.
